# Specificity and Evolvability in Eukaryotic Protein Interaction Networks

**DOI:** 10.1371/journal.pcbi.0030025

**Published:** 2007-02-16

**Authors:** Pedro Beltrao, Luis Serrano

**Affiliations:** European Molecular Biology Laboratory, Structures and Computational Biology Program, Heidelberg, Germany; Spanish National Cancer Research Centre, Spain

## Abstract

Progress in uncovering the protein interaction networks of several species has led to questions of what underlying principles might govern their organization. Few studies have tried to determine the impact of protein interaction network evolution on the observed physiological differences between species. Using comparative genomics and structural information, we show here that eukaryotic species have rewired their interactomes at a fast rate of approximately 10^−5^ interactions changed per protein pair, per million years of divergence. For Homo sapiens this corresponds to 10^3^ interactions changed per million years. Additionally we find that the specificity of binding strongly determines the interaction turnover and that different biological processes show significantly different link dynamics. In particular, human proteins involved in immune response, transport, and establishment of localization show signs of positive selection for change of interactions. Our analysis suggests that a small degree of molecular divergence can give rise to important changes at the network level. We propose that the power law distribution observed in protein interaction networks could be partly explained by the cell's requirement for different degrees of protein binding specificity.

## Author Summary

To understand how the cell performs the required biological functions and reacts to changes in the environment, scientists have been studying how cellular components interact. In recent years, new experimental methods have immensely increased our ability to map out these connections. However, it is important to keep in mind that biological systems are constantly evolving to cope with environmental changes. What then is the impact of the genomic variability brought by point mutations, segmental duplications, etc., on these interaction networks? We have tried here to quantify the rate by which protein interactions changed during the evolution of eukaryotic cells. According to the authors, about 0.5% to 3% of the interactions can change every million years. Also, protein properties, such as binding specificity (defined as the number of binding surfaces or binding partners) and protein function, help determine the rate of interaction turnover. This work suggests that protein interactions are evolutionarily plastic and the fact that a group of proteins has been conserved in different genomes does not mean that their interaction repertoire and functions are necessarily conserved. This work emphasizes the importance of studying biological systems in the context of evolutionary change.

## Introduction

Many partial protein interaction maps for several eukaryotic species have now been published [1–9], and several studies have tried to analyse the structure and evolution of such networks on a topological level [10–16]. The massive data produced by sequencing efforts has allowed for comparison of different genomes, giving us much more information about their organization and evolution than what would be possible by the analysis of individual genomes [17–21]. By analogy, to obtain a similar insight into the structure and evolution of interactomes, different protein networks would need to be compared, a procedure one could call “comparative interactomics.” This could be achieved by studying the conserved interactions between groups of ortholog proteins in different species, defined as interologs [1,22,23]. Recent attempts to compare protein interactions networks of different species [24,25] suggested that, at the moment, the current data are poorly suited for direct comparison; the overlap between datasets is small and this is most likely due to insufficient data quantity and quality. An alternative approach has been to look for conservation of network modules between species [26,27]. This has lead to the development of several network alignment tools and has largely been more successful at finding conserved interactions [26,28].

These studies have not, however, taken into account the large evolutionary distance separating the species under study. In fact, the four eukaryotic species for which we currently have the most interaction data (Saccharomyces cerevisiae, Drosophila melanogaster, *Caenorhabditis elegans,* and Homo sapiens) shared a common ancestor more than 900 million years (My) ago [29]. This raises the intriguing hypothesis that this evolutionary distance is too large for these species to have retained many similarities in their interaction networks. In fact, the relative success of the network alignment approaches hints that evolutionary pressures do not constrain the exact cellular interactions, but instead constrain the required functional complexes that emerge. Hence, the lack of overlap would correspond in part to divergence and not merely to poor coverage.

Even without the ability to compare interactomes directly, one could try to obtain estimates for the rate of change in interactomes by mining existing data with comparative genomics. Previously, Wagner [13,30] used paralogous protein pairs from S. cerevisiae to estimate that 2 × 10^−6^ new interactions are added to the yeast interactome per protein pair per My, suggesting that, on average, about 50 to 100 new interactions have arisen every 1 My. The method relied on very few interactions between paralogous protein pairs, making it impossible to dissect the different contributions to the average value. Also, in the last three years, we have more data available from a greater number of species, allowing us to compare how interactomes have changed in more recent evolutionary time and to draw more general conclusions about these processes.

We have attempted to evaluate the rate of change of interactions in the interactomes of several eukaryotic species (S. cerevisiae, D. melanogaster, *H. sapiens,* and C. elegans). We have determined that, in all species tested, interactions change at a rate on the order of 1 × 10^−5^ interactions per protein pair per My. The approach used allowed us to explore different protein properties influencing interaction turnover. We have observed that specificity in protein binding and protein function can determine link dynamics during the evolution of cellular networks.

## Results

### Recently Duplicated Proteins of Eukaryotic Species Have a Fast Rate of Interaction Change

To calculate the rate of interaction change, we have established for each protein, in all species studied here, an approximate age of origin, according to the presence or absence of an identifiable ortholog in several other reference species (see Materials and Methods). We assumed that any interaction observed today with a recently duplicated protein was either inherited in the process of duplication or created after the process of gene duplication. It has been estimated that for S. cerevisiae the fraction of gene duplicates with at least one shared interaction is below 20% after 50 My [13]. For each species, we calculated the fraction of interactions that were also observed with the closest homolog of the recently duplicated protein. Within the time frame studied (20 to 100 My), the fraction of observed conserved interactions after gene duplication was low (1% to 20%, see Tables 1 and S1). Eliminating the interactions likely inherited by duplication, we were left with protein interactions that were either gained in the copy we are considering or they were inherited by duplication and subsequently lost in the homolog. Either of these cases represents an event of interaction change that occurred after gene duplication, and we can thus calculate the rate at which interactions change (see Materials and Methods). This rate for all species was approximately on the order of 1 × 10^−5^ interactions changed per protein pair per My (see Table 1) and ranged from 5.36 × 10^−6^ in H. sapiens to 2.45 × 10^−5^ in S. cerevisiae. Considering the possible protein pairs in the different species (about 1.8 × 10^7^ in S. cerevisiae and 2.3 × 10^8^ in *H. sapiens,* when excluding splicing alternatives), this estimate would correspond to a change of approximately 100 to 1,000 interactions every My.

**Table 1:**
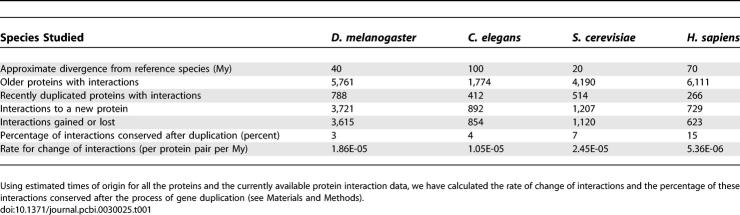
Eukaryotic Species Had in the Recent Evolutionary Past a Fast Rate of Change of Interactions

Due to the low coverage of the current interactomes, it is quite possible that these values might change as new data is made available. To study the impact of coverage on the values mentioned above, we have mimicked the effect of lowering the coverage of the current datasets by randomly sampling the interactomes in two ways: randomly removing protein interactions or randomly removing proteins (and their interactions).

The percentage of inherited interactions increased with increasing coverage; approximately linearly in the case of random node removal and nonlinearly in the case of random protein removal (see Figure S1). Therefore, the percentage of inherited interactions is a measure that depends on the size of the network and is therefore likely to change as more data is added.

The rate of change of interactions, on the other hand, appears to be independent of network size. There was no significant change in the rate in the case of random removal of proteins. In the case of random removal of interactions, only when more than 80% of the interactions were removed was there a significant increase of the rate (see Figure S1). It is important to note that the trend points to a stabilization of the value, indicating that further increases in coverage will not likely change it. We deduce from this result that the rate of change of interactions is a measure that is mostly independent of network size and can be used to study the evolution of protein interaction networks.

To test the robustness of our method for variations in accuracy of the data, the human interactome was separated into three subsets, as defined in the Human Protein Reference Database: (1) yeast two-hybrid (including the two recent high-throughput studies [3,4]); (2) in vitro studies, such as GST pull-down; and (3) in vivo studies, such as co-immunoprecipitation. The in vivo and in vitro subsets contain only literature-derived interactions and should therefore be of higher accuracy. The rate of change of interactions for these three datasets was 1.09 × 10^−5^, 7.23 × 10^−6^, and 7.12 × 10^−6^, respectively (see Table S1). The calculated rate using the high accuracy datasets was very similar to the values from the human yeast two-hybrid data and the data from the other species. We conclude that the order of magnitude reported is a good approximation for the rate of change of eukaryotic protein interactions networks.

### The Rate of Change of Interactions Correlates with the Number of Binding Partners of a Protein in All Eukaryotic Species Studied

Topological analysis of protein interaction networks has shown previously that the distribution of the number of interactions follows a power law, such that the frequency of proteins with n interactions falls off as n^−α^ (where alpha is the exponent of the power law) [15]. It has been proposed that the observed power law distributions could arise by network growth and preferential attachment, where newly created nodes would preferentially link to already highly connected nodes [31]. Two independent studies have shown, for S. cerevisiae, preferential attachment in the protein interaction network [11,30], supporting the model proposed for the emergence of the power law distribution (at least in the case of protein interaction networks).

**Figure 1:**
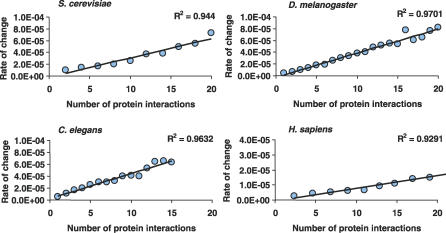
Preferential Interaction Turnover Is Observed in All Eukaryotic Interactomes We have binned proteins according to their average number of interactions and calculated for each bin the rate of change of interactions. There is a very strong correlation between the degree of connectivity and the interaction turnover.

We asked if there was also a correlation between the number of partners of a protein and the rate of change of its interactions. Given that the rate of change was calculated as the number of changed interactions per protein pair per My, no bias was expected for proteins having different degrees of connectivity. Using the data for different species, we observed a linear correlation between the number of binding partners and the link turnover for all species studied (Figure 1). The correlation in all cases is strong with R^2^ of 0.94 for *S. cerevisiae,* 0.97 for *D. melanogaster,* 0.96 for *C. elegans,* and 0.93 for H. sapiens. From these results we can conclude that there is preferential turnover of interactions in eukaryotic interactomes with highly connected proteins having a faster rate of change in their interactions.

### Domain Binding Characteristics Influence the Rate of Change of Interactions

The initial observations about the robustness of power law networks to random node removal [32] and the correlation between protein essentiality and the number of interactions [15] led to the idea that the overall structure of interactomes could be the outcome of selection for robustness to gene deletion. Wagner [30] suggested that there is no need to invoke natural selection to explain the network topology, since the local preferential attachment and network growth observed in the S. cerevisiae interactome suffice to create power law networks. We showed above that preferential turnover is a property of the protein interactions networks of all species studied, but what might determine this behavior at a molecular level?

It is known that interfaces of transient protein–protein interactions are less restricted in evolution than the binding surfaces of permanent complexes. Also, interacting residues of transient complexes are less likely to co-evolve than interaction residues of permanent complexes [33]. It is plausible that different types of interactions have different likelihoods of changing their interactions over a given evolutionary period. To test this in an unbiased fashion, we first aimed to determine the protein domains that contribute more to the fast rate of change. We considered only domains that conferred a rate of change to a group of proteins that was larger than average in at least three of the four species studied (see Materials and Methods). All of the domains selected by this criteria (see Table 2) are currently known to mediate protein–protein interactions. Interestingly, five of the eight domains are known to form domain–peptide interactions, as opposed to domain–domain interactions, suggesting that proteins containing peptide-binding domains are more likely to change their interactions faster over the same evolutionary time than other types of domains [34].

**Table 2:**
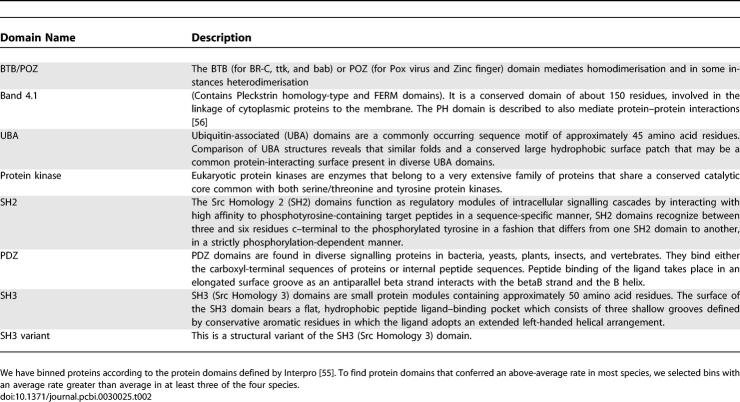
Domains Found to Contribute to the Fast Rate of Change of Interactions in at Least Three of the Four Species Studied

Many domain–peptide interactions involved a globular domain binding to a peptide that does not adopt a regular secondary structure and that is not part of the globular region of the target protein [35]. Since the interaction area is small, changes in the critical residues can easily abolish binding. Also, random point mutations in the proteome can more easily create new binding sites for these domains. This could explain their fast link turnover.

**Figure 2:**
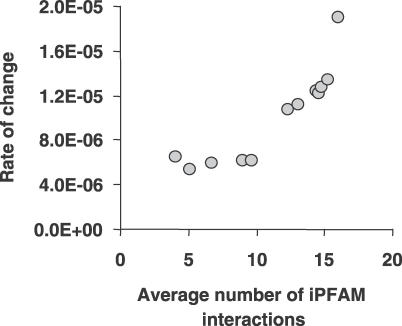
Protein Binding Domains with Many Structural Interactions Observed Have a Higher Link Turnover We have grouped proteins containing domains with increasing observed structural interactions with other domain types and calculated for each bin the rate of change of interactions. Proteins containing domains known to interact with many other different domains have a higher rate of change of interactions than proteins containing domains with few known interactions.

To test this hypothesis further, we analyzed a database containing structures of interacting protein domains [36] to search for domains with different degrees of specificity. Here, we define a “specific protein” as one that has few ligands and few interaction surfaces, while an “unspecific protein” is one that has many potential ligands and/or many different interaction surfaces. Therefore, promiscuous protein-binding domains are those that have been observed in close physical contact with a high number of other types of domains. We have binned proteins containing domains with increasing number of known structural interactions with other domains and calculated for each bin the rate of change (see Materials and Methods and Figure 2). Using the human dataset of literature-derived protein interactions, we observed that groups of proteins having domains capable of interacting with a higher number of other domains tend to have higher rates of change of their interactions.

For promiscuous proteins (containing domains capable of interacting with 15 or more other domains), we calculated the rate of change of interactions and compared this value with that obtained for proteins containing more specific domains (fewer than five structural interactions in iPfam) and the average for all proteins. We observed that peptide-binding domains and promiscuous domains have a higher rate of change of interactions (*p*-value < 0.02 for both groups with a Mann Whitney U test) than the average for the proteome (see Table 3). The difference between these two groups is not statistically significant (*p* = 0.06 with a Mann Whitney U test). Proteins having more specific binding domains have a rate of change of interactions not significantly different from the average value (*p* = 0.86 with a Mann Whitney U test).

**Table 3:**
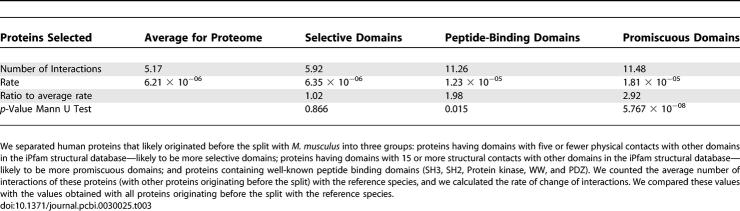
Specificity of Protein Binding Is an Important Factor Determining the Rate of Change of Interactions

We have also studied domains with different binding specificities as defined by their number of observed physical interactions. Using the iPfam [36] database, we were able to assign plausible binding interfaces to roughly 20% of human interactome (see Materials and Methods). We then searched for proteins that had multiple interactions occurring through the same domain, and we compared this group to another having proteins with a similar number of interactions through more than one domain (see Figure S2). Given the same number of interactions, proteins that preferentially bind through one domain (more promiscuous) have on average a higher rate of change of interactions than proteins interacting through multiple domains. For protein hubs having 10 to 50 interactions, the rate is 2.2 times higher for the promiscuous domains (*p*-value = 0.0089 with a Mann Whitney U test) than the more selective domains with the same average number of interactions.

We conclude from these results that the specificity, as defined by the number of binding surfaces or physical interactions, of a binding domain is a strong determinant of the rate of change of interactions, with more promiscuous binding correlating with higher rates of evolution.

### Human Proteins Related to Immune Response, Transport, and Localization Have Likely Been Under Positive Selection for Change of Their Interactions in the Recent Evolutionary Past

**Figure 3:**
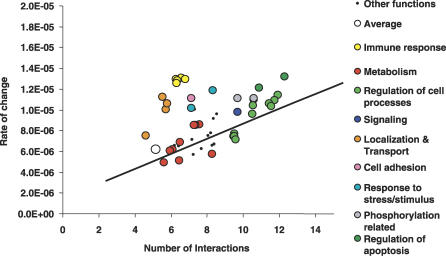
Biological Process Can Determine Link Dynamics Independently of the Number of Interaction Partners We have binned proteins according to the biological processes, defined in Gene Ontology, and calculated for each bin the average number of interactions and average rate of change of interactions (see Materials and Methods). The black line represents the expected rate of change for proteins with increasing numbers of interactions.

The results above suggest that specificity, as defined here, determines link dynamics, affecting the rate at which proteins might explore possible beneficiary interactions and remove deleterious ones. Innovation explored in this way is grounds for natural selection to act upon during evolution. It is then plausible that proteins belonging to different functional classes might have different rates of change of interactions due to differential selection pressures. To study this, we have binned human proteins according to Gene Ontology biological processes [37] (see Materials and Methods) and have, for each biological process, calculated the average number of interactions and the rate of change (see Figure 3). To reduce the number of functions and increase the reliability of the calculated rates, we have considered only GO processes with at least 150 proteins present in the current human interactome (excluding yeast two-hybrid interactions). We compared the values obtained with the expected rates for proteins with increasing average connectivity (black line in Figure 3).

Most groups of proteins had average rates of change that were not much different than groups of proteins with a similar connectivity. Within these groups we could distinguish between processes that have proteins with similar or lower-than-average rates (such as metabolism) and biological processes that have above-average rates (such as intracellular signaling, phosphorylation, regulation of cellular processes, and regulation of apoptosis). These results confirm the suggestion of Kunin et al. [38] that the functional role of proteins poses constraints on the evolution of new interactions. We suggest this is due to differential usage of protein binding specificity in the different cellular processes.

More interestingly, we found some biological processes (immune response, transport and localization, cell adhesion, and response to stress/stimulus) that showed higher link dynamics than one would expect from their average number of interactions. On Table 4 we list the biological processes that have a rate significantly higher (*p*-value < 0.05 with a Mann Whitney U test) than expected from their average connectivity.

**Table 4:**
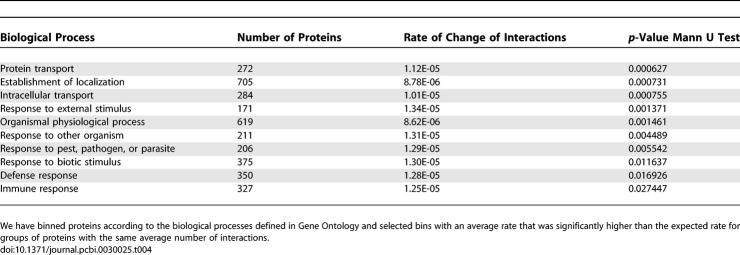
Biological Processes with Above-Average Rate of Change of Interactions

We redid this analysis removing GO annotations inferred electronically. Although roughly 50% of the annotations were lost, most of the results remain qualitatively the same (unpublished data). Importantly, we still see GO functions that have a rate significantly higher than expected from their average connectivity (organismal physiological process, defense response, immune response, and response to biotic stimuli). We hypothesize that the groups of proteins deviating from the linear preferential turnover have been under particularly strong positive selection for the change of their interactions.

## Discussion

In the seminal work of King and Wilson [39] (“Evolution at two levels in humans and chimpanzees”), it was proposed that, given the small differences observed between the proteins in both species, the most likely mechanism that could account for the differences between humans and chimpanzees would be changes in gene expression. In fact, in the 30 years following, a considerable amount of evidence has surfaced to support this view (for a review see [40]). However, as we have shown above, it is quite possible that in the time since the divergence of these two species, a significant change in protein interactions could have taken place, and, therefore, changes in protein interaction networks can have a similar important role in species evolution.

### Calculation of the Rate of Change and Potential Caveats

Extending on the work of Wagner [13,30] for *S. cerevisiae,* we have shown here that the interactomes of several eukaryotic species have high rates of change of their interactions. According to our calculations, 100 to 1,000 interactions might change every My in eukaryotic interactomes. Estimating that in the current interactomes proteins have on average four to ten interactions, then the link turnover would change 0.5% to 3% of the interactions every My. This estimate clearly points to an important effect of link dynamics in the evolution of cellular interactions.

Some caveats to our estimated rate should be noted. Namely, we have focused our attention on the evolution of protein interactions of single gene duplicates. The effects of single gene duplication could be considerably different from large segment or whole-genome duplication events [41]. If a protein that is part of a complex duplicates, the changes in gene dosage might unbalance the complex stoichiometry and cause a reduction in fitness to the species [42]. On the contrary, if the whole complex is duplicated, as would occur in a full-genome duplication event, the balance between the complex components would be maintained. We can speculate that a single-gene duplicate might have a stronger pressure to diverge in its interactions to avoid a gene imbalance effect, when compared with proteins originated from a full-complex duplication. However, it is also known that living systems have mechanisms to guarantee the right expression level when dealing with important complexes. A good example is the case of tubulin where the stability of the mRNA depends on the existence of unpaired α or β subunits [43]. In fact, it is almost impossible to keep the right stoichiometry in a complex simply due to noise fluctuations, unless there are mechanisms that control possible imbalances. Thus, although it is intuitive that whole-genome duplication could exert less pressure for divergence, this does not necessarily need to be true.

Also, it has been proposed that duplicate genes pass through a period of relaxed selection after gene duplication [21] possibly accompanied by a relaxation in the selection for maintenance of their interactions. As a result of this, the link dynamics are likely higher for recently duplicated genes than for proteins with established functions. However, experimental studies estimated that 69% to 84% of interactions between ancient proteins have diverged when comparing S. cerevisiae with C. elegans [22]. The authors tested 216 interactions that were known from S. cerevisiae among 282 C. elegans proteins (that are conserved in S. cerevisiae). Of these, 35 interactions were experimentally observed in C. elegans. We can estimate that approximately 5 × 10^−6^ interactions have changed per protein pair per My among these ancient proteins. This is roughly half of what we proposed as an estimate for the rate of change for eukaryotic interactomes, but it considers only removal of interactions and not addition. Due to the reasons mentioned above, we think that the rate that we have calculated might be an overestimate of the rate for the whole interactome, but currently available data suggests that even among ancient proteins there is considerable change of their interactions.

Other studies also point to the importance of change of interactions after gene duplication. In studies of S. cerevisiae complexes, it was observed that 7% to 20% of those complexes arose by duplication [44,45]. However, in most of the instances (67% to 90%), only partial duplication was observed. Detailed studies of some of the duplicated complexes have shown that even in the case where general function and localization is maintained, it is possible that the specific activity of the complex has changed. These results suggest that, at least in the case of protein complexes, there is a very significant gain and loss of single interactions that can lead to the formation of new complexes with different specificities.

### Binding Properties and Function Determine the Rate of Change

In a recent review [34], Neduva and Russell postulated that linear motifs might act as “evolutionary interactions switches.” The authors claimed that, due to binding to a linear peptide stretch and due to the small number of determinants for binding, a random mutation in the proteome can easily create or destroy such binding sites. We have shown that specificity of binding is a key factor determining the evolvability of the interaction networks. More promiscuous domains, such as peptide binding domains and domains able to interact with common structural elements, are more likely to change interactions faster over a given evolutionary period. This implies that some biological processes where these binding domains are involved (such as signal transduction) will also have a higher link turnover than others (such as metabolism). In fact, proteins related to intracellular signaling cascades are two times more likely to interact with recently duplicated proteins than proteins involved in primary metabolism. If protein properties impact on how they add and lose interactions, then the power law distribution observed in protein interaction networks is in part determined by the cell's use of different degrees of binding specificity.

We reported that proteins involved in the immune response, responses to external stimuli, transport, establishment of localization, and organismal physiological processes show signs of such positive selection for new interactions. Interestingly, most of these biological processes are known to have an excess of proteins under positive selection as shown by sequence studies [46,47]. Our results suggest that positive selection for sequence change in proteins is propagated to faster rates of change at the network level. Alternatively, it is possible that the preferential fixation of duplicated proteins involved in these biological functions might partly explain this observation.

### Link Dynamics and Cellular Evolution

This study opens up interesting questions regarding the evolution of cellular functions. Some challenges faced by the cells require the interaction of several components to integrate information and provide a solution. One example would be the decision to divide or differentiate given a set of external conditions. It could be said that these challenges require a network solution as opposed to some metabolic problems, such as adapting an enzyme to do a required metabolic step.

In network challenges as defined above, selection forces would not restrain the exact binary interactions, but rather the functional complexes arising from them. It is plausible that the fast link dynamics are then advantageous to the cell, given that it allows for exploration of different network conformations from where innovation might arise.

If there is indeed a fast turnover of interactions that are material for selection to act upon, then we expect to see convergent network motifs that are optimal for solving particular cellular problems. An example of what might be an optimal network solution is the coupling of slow and fast positive feedbacks in cell decision processes [48].

If fast link dynamics is important for the cell to search for optimal solutions to network problems, then is it also likely that the rate of change itself might be under constraint and therefore under natural selection. Hence, during cellular evolution, the selection of different degrees of specificity is not only important for the functional role of the proteins but it also has direct consequences with regard to the evolvability of the whole cellular network.

Further work on protein interaction maps will help us understand to what extent evolvability constrains the differential usage of protein domains in cellular networks. As was the case for comparative genomics, the availability of more and complete interactomes for different species will vastly increase our understanding of how the cell's complexity arises from the interactions of its components and evolves to cope with changing environments.

## Materials and Methods

### Estimating protein time of origin.

For each protein of *S. cerevisiae, D. melanogaster, C elegans,* and *H. sapiens,* we have established a likely time of origin by looking for putative orthologs in several other fully sequenced genomes (that we refer to here as reference species) using the orthologs defined in the Inparanoid database (http://inparanoid.sbc.su.se [49]). For the species not covered in the Inparanoid database, we have used the reciprocal BLAST best-hit method. Succinctly, we considered that if two proteins were the reciprocal best blast hit between two species, then these proteins are likely orthologs.

We established putative orthologs between S. cerevisiae and the following species: *S. bayanus, Candida glabrata, Kluyveromyces lactis, Asphis gossypii, C. albicans, Debaryomyces hansenii, Yarrowiya lipolytica, Neurospora crassa, Schizosaccharomyces pombe*. For *D. melanogaster,* we used as reference species D. pseudoobscura, Anolpheles gambiae, *C. elegans,* and S. cerevisiae. For H. sapiens proteins we have looked for putative orthologs in Mus musculus, Fugu rubripes, *D. melanogaster,* and S. cerevisiae. For C. elegans proteins, we have searched for putative orthologs in C. briggsae, *D. melanogaster,* and S. cerevisiae.

We considered that proteins with no apparent ortholog in any of the reference species likely originated after the divergence of the most recently diverged reference species. S. cerevisiae proteins with no putative ortholog in any of the reference species most likely originated after the divergence of S. cerevisiae and S. bayanus, ~20 My ago [50]. D. melanogaster proteins with no apparent ortholog are younger than the split with D. pseudoobscura (~40 My ago) [51]. C. elegans proteins with no apparent ortholog are younger than the split with C. briggsae (~100 My ago) [52]. H. sapiens proteins with no apparent ortholog is younger than the split with M. musculus (~70 My ago) [53].

### Calculating the rate of change of interactions.

The interactomes used for C. elegans, *S. cerevisiae,* and D. melanogaster were extracted from BIND (http://bind.ca [54]), including only direct protein–protein interactions. To create the human interactome used here, we added protein interactions from two recent studies of yeast two-hybrid studies [3,4] to the literature-derived interactions compiled in the human protein reference database (taking only direct protein–protein interactions) [2].

We considered that an interaction was inherited in the process of duplication when an interaction to a recently duplicated protein was also observed with its closest homolog. Removing these interactions we were left with protein interactions that were either gained in the copy we are considering or were inherited by duplication and subsequently lost in the homolog. Either of these cases represents an event of interaction change that occurred after the gene duplication event.

The rate of change of interactions can be calculated by: rate = changed interactions/(possible protein pairs * divergence time). Designating the recently duplicated proteins as P_new_ and proteins originated before the split with the most recently diverged reference species as P_old_, then: changed interactions = changed interactions among P_new_ + changed interactions between P_new_ and P_old_. Possible protein pairs = P_new_ * P_old_ + (P_new_ * (P_new_ − 1)/2). Divergence time = divergence time of the most recently diverged reference species (see above).

### Preferential interaction change, protein domains, and biological processes.

To determine if the number of interaction partners of a protein correlates with the rate of change of interactions, we have binned all proteins in P_old_ according to the number of interactions to other proteins in P_old_. We considered bins of proteins with i to i + 5 interactions, with i ranging from one to 20. For each of these bins, P_old(bin)_, the rate of change of interactions was considered to be: rate of change_ (bin)_ = changed interactions between P_new_ and P_old(bin)_/(P_old(bin)_ × P_new_ × divergence time).

We observed a very strong preferential turnover in all species such that proteins with a higher degree of connectivity have a higher rate of change. For proteins with k interactions, the rate of change, r, can be calculated by:

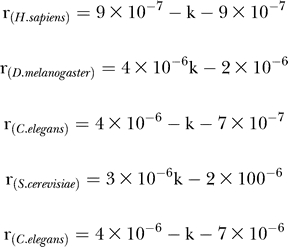
To determine which domains correlated with higher rates of change of interactions, we binned proteins in P_old_ according to the protein domains defined by Interpro [55]. For each protein domain, we retrieved all proteins in P_old_ where this domain was observed and calculated the rate of change of interactions as above. To select protein domains that conferred an above-average rate in most species, we selected bins with at least 20 proteins (to increase the reliability of the calculated rate) and with a rate greater than average in at least three of the four species. There are 96 protein domains that are represented by 20 or more proteins in at least three species. Of these, eight have an above average rate of change in at least three of the four species studied. The effect of both constrains (number of proteins and average rate) is detailed in Table S2.

To study protein domains with different binding specificity, we binned proteins containing domains with increasing number of interactions with other domains (extracted from the iPfam database) and calculated for each bin the rate of change. We considered bins of proteins in P_old_ having proteins domains with i to i + 10 iPfam interactions with i ranging from one to 15. We calculated the rate of change_ (bin)_ as above.

We have used the iPfam database to search for plausible binding interfaces in all human interactions derived from the human protein reference database. We could assign a possible binding interface to ~20% of the human interactome. We then built two groups of proteins according to the number of binding interactions per domain. We selected a group of proteins that had three or more interactions through one domain (likely more promiscuous domains), and a second group of proteins that interacted with three or more partners via multiple domains (likely more selective domains). We then further subdivided the two groups into bins protein with i to i + 5 interactions with i ranging from five to 15 and calculated the rate_ (bin)_ as above (see Figure S1).

To study the different biological processes, we have binned proteins in P_old_ according to the biological processes defined in Gene Ontology [37] (downloaded from http://www.geneontology.org on 29 January 2006). For each Gene Ontology biological process, we have selected all proteins defined as participating in that biological process or any of its GO term children processes. For all such bins of proteins, we have calculated the rate of change of interactions as above.

## Supporting Information

Figure S1 Dependence of the Calculated Rate of Change and Fraction of Conserved Interactions After Duplication on Network Size (A,B) Sampling of S. cerevisiae interactome. (C,D) Sampling of D. melanogaster interactome. (E,F) Sampling of H. sapiens interactome. (G,H) Sampling of C. elegans interactome.
(A,C,E,G) Sampling was done by randomly removing interactions. Any protein with no interaction is no longer considered as part of interactome.
(B,D,F,H) Sampling was done by randomly removing proteins and their interactions. Any protein with no interaction is no longer considered as part of interactome. Filled squares (▪), rate of change of interactions. Open squares (□), fraction of interactions conserved after duplication.
(145 KB PPT)

Figure S2 Specificity of Binding Can Determine the Rate Change of Interactions in Groups of Proteins with the Same Connectivity (A) We binned all proteins according to the number of interactions with proteins originated before the split with the reference species and calculated the rates of change of interactions with recently duplicated proteins.
(B) We mapped the most likely interacting domains in the human interactome using the database of interacting motifs. We selected two groups of proteins: blue circles (•), proteins having three or more interactions through at least two or more domains; red circles (•), proteins having three or more interactions through the same domain. We binned both groups according to the number of interactions occurring in the full interactome with proteins originated before the split with the reference species and calculated the rates of change of interactions with recently duplicated proteins.
(40 KB PPT)

Table S1 The Estimated Rate Calculated Is Robust to Variation in Accuracy of the Datasets Used To test for a possible bias of the experimental method used in determining protein interactions, we divided the interactions of the human dataset into three subsets, as defined in the Human Protein Reference Database: yeast two-hybrid, in vitro studies such as GST pull-down, and in vivo studies such as co-immunoprecipitation. The estimated rate of change of interactions calculated with the yeast two-hybrid method (including human high-throughput studies) was only marginally higher than those observed with the other two datasets (obtained exclusively from literature-derived protein interactions).
(31 KB DOC)

Table S2 Impact of Constraining the List of Domains by Representation in the Difference Species and by the Average Rate of Change To increase the reliability of the rate of change for each domain, we have selected only domains that were represented in most species by at least 20 domains. Of all Interpro domains, 96 observe this condition. Of these 96 domains, eight have an above average rate of change for at least three species studied. We can say that these eight domains consistently contribute to the fast rate of change in most species.
(26 KB DOC)

## Abbreviations

My: million years
UBA: ubiquitin-associated
