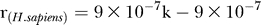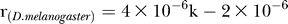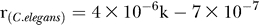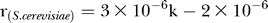# Correction: Specificity and Evolvability in Eukaryotic Protein Interaction Networks

**DOI:** 10.1371/journal.pcbi.0030070

**Published:** 2007-03-30

**Authors:** Pedro Beltrao, Luis Serrano

In *PLoS Computational Biology,* volume 3, issue 2: doi:10.1371/journal.pcbi.0030025

The set of four equations in the Materials and Methods section had three extra minus signs, which were incorrect. The following are the correct equations.