# Supplementary material for: Specificity and Evolvability in Eukaryotic Protein Interaction Networks
Source: PLoS Comput Biol. 2007 Feb 16;3(2):e25. doi: 10.1371/journal.pcbi.0030025 (PMC1797819; doi:10.1371/journal.pcbi.0030025)
Supplement: Supplementary file 1 [file pcbi.0030025.st001.doc]

Table S1 – **The estimated rate calculated is robust to variation in accuracy of the datasets used**. To test for a possible bias of the experimental method used in determining protein interactions, we divided the interactions of the human dataset into three subsets, as defined in the Human Protein Reference Database: yeast two-hybrid, in vitro studies such as GST pull-down and in vivo studies such as co-immunoprecipitation. The estimated rate of change of interactions calculated with the yeast two-hybrid method (including human high-throughput studies) was only marginally higher than those observed with the other two datasets (obtained exclusively from literature derived protein interactions).

|  | ***H.sapiens*** | ***H.sapiens*** | ***H.sapiens*** |
| --- | --- | --- | --- |
| Approximate divergence from reference species (My) | 70 | 70 | 70 |
| Experimental method | Yeast two-hybrid | *in vivo* | *in vitro* |
| Old proteins with interactions | 2693 | 3806 | 4021 |
| Recently duplicated proteins with interactions | 138 | 109 | 119 |
| Interactions between the two groups of proteins | 293 | 262 | 305 |
| Interactions gained or lost | 291 | 213 | 242 |
| Percentage of interactions conserved after duplication (%) | 1 | 19 | 21 |
| Rate of change of interactions (per protein pair per My) | 1.09E-05 | 7.23E-06 | 7.12E-06 |
