# Supplementary material for: Specificity and Evolvability in Eukaryotic Protein Interaction Networks
Source: PLoS Comput Biol. 2007 Feb 16;3(2):e25. doi: 10.1371/journal.pcbi.0030025 (PMC1797819; doi:10.1371/journal.pcbi.0030025)
Supplement: Supplementary file 2 [file pcbi.0030025.st002.doc]

Table S2 – **Impact of constraining the list of domains by representation in the difference species and by the average rate of change**. To increase the reliability of the rate of change for each domain we have selected only domains that were represented in most species by at least 20 domains. Of all Interpro domains, 96 observe this condition. Of these 96 domains, 8 have an above average rate of change for at least 3 species studied. We can say that these 8 domains consistently contribute to the fast rate of change in most species.

|  | In >=1 species | In >=2 species | In >=3 species | In 4 species |
| --- | --- | --- | --- | --- |
| Domains represented in at least 20 proteins | 628 | 189 | 96 | 12 |
| Domains represented by at least 20 proteins and rate of change above the average for the proteome | 271 | 57 | 8 | 1 |
